# The Synergistic Effects of Sulfated Lactosyl Archaeol Archaeosomes When Combined with Different Adjuvants in a Murine Model

**DOI:** 10.3390/pharmaceutics13020205

**Published:** 2021-02-02

**Authors:** Yimei Jia, Bassel Akache, Gerard Agbayani, Vandana Chandan, Renu Dudani, Blair A. Harrison, Lise Deschatelets, Usha D. Hemraz, Edmond Lam, Sophie Régnier, Felicity C. Stark, Lakshmi Krishnan, Michael J. McCluskie

**Affiliations:** 1 Human Health Therapeutics, National Research Council Canada, Ottawa, ON K1A 0R6, Canada; yimei.jia@nrc-cnrc.gc.ca (Y.J.); bassel.akache@nrc-cnrc.gc.ca (B.A.); gerard.agbayani@nrc-cnrc.gc.ca (G.A.); vandana.chandan@nrc-cnrc.gc.ca (V.C.); renu.dudani@nrc-cnrc.gc.ca (R.D.); blair.harrison@nrc-cnrc.gc.ca (B.A.H.); lise.deschatelets@nrc-cnrc.gc.ca (L.D.); felicity.stark@nrc-cnrc.gc.ca (F.C.S.); lakshmi.krishnan@nrc-cnrc.gc.ca (L.K.); 2 Aquatic and Crop Resource Development, National Research Council Canada, Montreal, QC H4P 2R2, Canada; usha.hemraz@nrc-cnrc.gc.ca (U.D.H.); edmond.lam@nrc-cnrc.gc.ca (E.L.); sophie.regnier@nrc-cnrc.gc.ca (S.R.)

**Keywords:** archaeosome, vaccine adjuvant, glycolipid, sulfated lactosyl archaeol

## Abstract

Archaeosomes, composed of sulfated lactosyl archaeol (SLA) glycolipids, have been proven to be an effective vaccine adjuvant in multiple preclinical models of infectious disease or cancer. SLA archaeosomes are a promising adjuvant candidate due to their ability to strongly stimulate both humoral and cytotoxic immune responses when simply admixed with an antigen. In the present study, we evaluated whether the adjuvant effects of SLA archaeosomes could be further enhanced when combined with other adjuvants. SLA archaeosomes were co-administered with five different Toll-like Receptor (TLR) agonists or the saponin QS-21 using ovalbumin as a model antigen in mice. Both humoral and cellular immune responses were greatly enhanced compared to either adjuvant alone when SLA archaeosomes were combined with either the TLR3 agonist poly(I:C) or the TLR9 agonist CpG. These results were also confirmed in a separate study using Hepatitis B surface antigen (HBsAg) and support the further evaluation of these adjuvant combinations.

## 1. Introduction

Adjuvants are key components of vaccine formulations, providing the immune stimulation necessary to generate effective immune responses against poorly immunogenic antigens. While only a small number of vaccine adjuvants are currently licensed for use in human vaccines [1,2], novel adjuvant formulations could prove useful in increasing the activity/efficacy of experimental vaccines, in particular for those requiring strong cell-mediated immune responses [3]. While many subunit vaccines originally contained only single adjuvants (e.g., aluminum salts and oil-in-water emulsions such as MF59), a combination of adjuvants with different mechanisms of action can further enhance immunogenicity. Indeed, of the currently approved vaccine adjuvants, several contain multiple immunostimulatory components. These include the Adjuvant Systems AS01 (a liposome-based vaccine adjuvant system containing the Toll-like receptor 4 (TLR4) agonist 3-O-desacyl-4ʹ-monophosphoryl lipid A (MPLA) and the saponin QS-21) and AS04 (a combination of the TLR4 agonist MPLA and aluminum hydroxide) [4]. There is also strong pre-clinical data to support other adjuvant combinations, e.g., the TLR9 agonist CpG together with aluminum hydroxide [5,6], ISCOMs [7], liposomes [8,9] or oil-in-water emulsions [10] and the TLR3 agonist poly(I:C) in combination with aluminum hydroxide [5] to name but a few.

Archaeosomes are a type of liposome traditionally comprised of total polar lipids (TPL) or semi-synthetic glycerolipids of ether-linked isoprenoid phytanyl cores with varied glyco- and amino-head groups. They have been used as a vaccine adjuvant in pre-clinical studies for many years and have been shown to promote strong humoral and cell-mediated responses to entrapped antigens. Archaeosome-based formulations were able to protect immunized mice from multiple pathogens including *Listeria monocytogenes*, *Trypanosoma cruzi* and *Mycobacterium tuberculosis*, as well as to protect against solid and metastatic tumors in murine models [11,12,13,14]. More recently, we showed that a simplified archaeosome formulation composed of sulfated lactosyl archaeal (SLA) glycolipid can stimulate strong humoral and cell-mediated immune responses to multiple antigens in mice, including those targeting various infectious pathogens (influenza virus, Hepatitis B virus, Hepatitis C virus and *Schistomiasis mansoni*) [15,16,17,18] or tumor types (breast cancer and melanoma) [19,20]. While originally used as delivery vehicles for encapsulated antigen, a simplified SLA formulation where antigens were simply admixed with pre-formed empty SLA vesicles was shown to generate equivalent or superior humoral and cell-mediated immune responses to conventional antigen-entrapped archaeosome formulations [21]. Although the mechanism of action of the admixed SLA archaeosome formulation has not been fully elucidated, it does increase immune cell infiltration, antigen retention at injection site and antigen uptake by antigen-presenting cells and other immune cell types, including neutrophils and monocytes [22,23]. 

Herein, we evaluate the potential of SLA archaeosomes to synergize with various known immunostimulants, including TLR agonists and the saponin QS-21. Humoral and cell-mediated immune responses to the model antigen ovalbumin (OVA) were evaluated following vaccination using SLA archaeosomes as adjuvant alone or in combination with the TLR1/2 agonist Pam_3_CSK_4_, the TLR3 agonist poly(I:C), the TLR4 agonist MPLA, the TLR 7/8 agonist R848, the TLR9 agonist CpG or the saponin QS-21. The synergistic adjuvant activity of the most potent combinations, i.e., SLA + Poly(I:C) and SLA + CpG, was then confirmed using Hepatitis B surface antigen (HBsAg).

## 2. Materials and Methods

### 2.1. Materials

Sulphated lactosyl archaeol (SLA; 6′-sulfate-β-D-Gal*p*-(1,4)-β-D-Glc*p*-(1,1)-archaeol) was synthesized as described previously [24]. Archaeosomes were prepared as previously described [21]. Briefly, 30 mg of SLA lipid was dissolved in chloroform/methanol; a thin film was formed after removal of solvent under N_2_ gas with mild heating. A vacuum was applied to ensure total removal of trace solvents. Dried lipids were hydrated in 700 µL of Milli-Q water without protein antigen. Lipid dispersions were shaken for 2–3 h at 40–50 °C until completely suspended. Next, a brief sonication was applied at 40 °C in an ultrasonic water bath (Fisher Scientific, Ottawa, ON, Canada) for up to 60 min until the desired particle size (between 100 and 200 nm) was obtained. Approximately 300 µL of 10× PBS (Millipore Sigma Canada, Oakville, ON, Canada) was added to balance osmolality and reach pH of 7.4. The pre-formed empty SLA archaeosomes were stored at 4 °C at a concentration of 30 mg/mL until used.

All TLR agonists (i.e., CpG, MPLA, Pam_3_CSK_4_, Poly(I:C), R848) were purchased from InvivoGen (San Diego, CA, USA), while QS-21 was purchased from Desert King (San Diego, CA, USA). Stock solutions were prepared according to the manufacturer’s instructions, at 1 mg/mL for CpG, MPLA, Pam_3_CSK_4_, Poly(I:C), R848 and QS-21. Antigen stock solutions for ovalbumin protein (ovalbumin; OVA; type VI, Sigma-Aldrich, St. Louis, MO, USA) and recombinant HBsAg (HBsAg; Subtype adw; Fitzgerald Industries International, Acton, MA, USA) were prepared at a concentration of 1 mg/mL. 

### 2.2. Animals 

The 6–8 week old female C57BL/6 and BALB/c mice in this study were obtained from Charles River Laboratories (Saint-Constant, QC, Canada) and used for OVA and HBsAg immunization, respectively. Animals were monitored for adverse clinical signs (such as piloerection, dehydration, hunched posture, labored breathing and reduced mobility) immediately following vaccination and routinely throughout the course of the study.

All experimental procedures were conducted in accordance with the guide for care and use of laboratory animals, and the animal procedures were performed in accordance with the Ethics Committee of the National Research Council of Canada with approval certificate registration number 2016.08 (approved August, 2016), and followed the recommendations of the National Institutes of Health Guide for Care and Use of Laboratory animals.

### 2.3. Vaccine Formulation and Immunization

The combination adjuvant formulations were prepared by first mixing empty pre-formed SLA archaeosomes with an additional adjuvant (Poly(I:C), CpG, MPLA, R848, Pam_3_CSK_4_ or QS-21) and briefly vortexing. Thereafter, OVA or HBsAg antigen solution was added and briefly vortexed. Finally, PBS buffer was added to dilute solutions to the required concentration for immunization. The final dose administered for each component in OVA and HBsAg vaccine formulations is indicated in Table 1 and Table 2, respectively. Mouse strains, antigen and adjuvant doses for each antigen were based on previous studies conducted in our laboratories or based on the manufacturer’s recommendation. However, in an effort to detect synergistic responses without reaching the limits of detection of our assays, suboptimal dose levels (2-fold lower than used previously) for both antigen and adjuvant were used in the OVA vaccine studies. As cellular responses with 2 µg HBsAg are generally lower (unpublished observations), we used the full adjuvant dose amounts in that study.

Mice were immunized by IM injection (50 µL) into the left tibialis anterior (T.A.) muscle on days 0 and 21 with a total dose of 5 µg OVA or 2 µg HBsAg alone or formulated with the various adjuvants as described above. Animals were bled on day 28, and recovered serum was used for quantification of antigen specific IgG antibody levels. For collection of serum, blood was allowed to clot for at least 30 min in serum separating tubes (Becton Dickinson, Franklin Lakes, NJ, USA) prior to centrifugation for 15 min at 3500× *g*. On day 27, carboxyfluorescein succinimidyl ester (CFSE)-stained target cells (as described below) diluted in Hank’s balanced salt solution (HBSS; GE Life Sciences, Chicago, IL, USA) to a final volume of 200 μL were injected into the retro-orbital plexus to assess antigen-specific in vivo cytolytic killing. Spleens were collected on day 28 for elucidation of cellular immune responses by IFN-γ ELISpot and/or in vivo cytolytic activity assay.

### 2.4. Pathogen Recognition Receptors Stimulation Assay

To determine the ability of SLA to activate various Pattern Recognition Receptors (PRR), we utilized Invivogen’s PRR ligand screening service. Toll-like receptor (TLR) and NOD-like Receptor (NLR) stimulation was assessed through NF-κB activation in HEK293 cells expressing a given TLR or NLR. NF-κB controls the expression of secreted embryonic alkaline phosphatase (SEAP) reporter gene. In a 96-well plate (200 μL total volume) containing the appropriate cells (50,000–75,000 cells/well), 20 μL of the test article or the positive control ligand was added to the wells. The media added to the wells are designed for the detection of NF-κB-induced SEAP expression. After a 20 h incubation, the optical density (OD) was read at 650 nm on a SpectraMax 340PC absorbance detector (Molecular Devices, San Jose, CA, USA).

SLA at concentrations of 150 and 50 µg/mL was tested in triplicate on cell lines expressing eight different mouse TLRs (TLR2, 3, 4, 5, 7, 8, 9 and 13) and two different mouse NLRs (NOD1 and NOD2). Different positive control ligands were included for each of the various PRR cell lines: (1) mTLR2: heat-killed *Listeria monocytogenes* (HKLM) at 1 × 10^8^ cells/mL, (2) mTLR3: Poly(I:C) HMW at 1 μg/mL, (3) mTLR4: *E. coli* K12 LPS at 100 ng/mL, (4) mTLR5: *S. typhimurium* flagellin at 100 ng/mL, (5) mTLR7: CL307 at 1 μg/mL, (6) mTLR8: CL075 at 10 μg/mL + Poly(dT) at 10 μM, (7) mTLR9: CpG ODN 1826 at 1 μg/mL, (8) mTLR13: ORN Sa19 at 200 ng/mL, (9) mNOD1: C12-iE-DAP at 1 μg/mL and (10) mNOD2: L18-MDP at 100 ng/mL. As negative controls, corresponding cell lines that do not express the above-mentioned PRRs were also stimulated with SLA and shown to have minimal SEAP expression.

### 2.5. Anti-OVA/HBsAg Antibody ELISA

The levels of anti-OVA or anti-HBsAg antibodies (Ab) in mouse serum were quantified by ELISA using a previously described method [15]. Briefly, 96-well high-binding ELISA plates were coated with OVA and/or HBsAg overnight. Plates were washed and then blocked with fetal bovine serum in PBS or carbonate/bicarbonate buffer for OVA and HBsAg, respectively. After the plates were washed, serial diluted samples were added in 100 µL volumes and incubated for 1 h at 37 °C. After five washes with PBS/0.05% Tween 20, 100 µL of goat anti-mouse IgG-HRP, goat anti-mouse IgG1-HRP or goat anti-mouse IgG2c-HRP was added for 1 h at 37 °C. Then the substrate o-phenylenediamine dihydrochloride was added. Plates were developed for 30 min at RT in the dark. Titers for IgG in serum were defined as the dilution that resulted in an absorbance value (OD 450) of 0.2 and were calculated using XLfit software (ID Business Solutions, Guildford, UK). 

### 2.6. ELISpot Assay

Enumeration of antigen-specific IFN-γ secreting cells was performed by ELISpot assay as previously described [15,17,21]. Briefly, spleen cells (at a final cell density of 4 × 10^5^/well) were added to ELISPOT plates coated with an anti-IFN-γ antibody (Mabtech Inc., Cincinnati, OH, USA), and incubated in the presence of appropriate antigen-specific stimulant at a concentration of 2 µg/mL for 20 h at 37 °C, 5% CO_2_. For OVA protein-immunized animals, CD8 T cell epitope OVA_257–264_: SIINFEKL or CD4 T cell epitope OVA_323–339_: ISQAVHAAHAEINEAGR peptides (JPT Peptide Technologies GmbH, Berlin, Germany) were used as stimulants. Cells were also incubated without any stimulants to measure background responses. The plates were then incubated, washed and developed according to the manufacturer’s instructions. AEC substrate (Becton Dickenson, Franklin Lakes, NJ, USA) was used to visualize the spots. Spots were counted using an automated ELISpot plate reader (BIOSYS, Miami, FL, USA). 

### 2.7. In Vivo Cytolytic Activity

In vivo cytolytic activity in immunized mice was enumerated as described previously [21,25]. Donor spleen-cell suspensions from syngeneic mice were prepared. Cells were split into two aliquots. One aliquot was incubated with the appropriate CTL specific peptide (10 μM; SIINFEKL for OVA experiments and IPQSLDSWWTSL for HBsAg experiments, JPT Peptide Technologies GmbH) in R10 media. After 30 min of incubation, the non-peptide containing aliquot was stained with a low concentration of CFSE (0.25 μM; Thermo Fisher Scientific, Waltham, MA, USA), and the second peptide-pulsed aliquot was stained with a 10-fold higher concentration of CFSE (2.5 μM). The two cell aliquots were mixed 1:1 and injected (total of 20 × 10^6^ cells/mouse) into previously immunized recipient mice. At ~20 to 22 h after the donor cell transfer, spleens were removed from recipients, single cell suspensions prepared and cells analyzed by flow cytometry on a BD Fortessa flow cytometer (Becton Dickenson, Franklin Lakes, NJ, US). The in vivo lysis percentage of peptide pulsed targets was enumerated according to equation in the reference above. 

### 2.8. Statistical Analysis

Data were analyzed using GraphPad Prism^®^ (GraphPad Software, San Diego, CA, USA). Statistical significance of the difference between three or more groups was determined by ANOVA followed by post-hoc analysis using Tukey’s multiple comparison tests. Antibody titers and ELISpot counts were log-transformed prior to statistical analysis. Outliers were identified by Grubbs’s test and removed. Differences were considered to be significant at *p* < 0.05.

## 3. Results

### 3.1. Activation of PRRs by SLA

**Figure 1:**
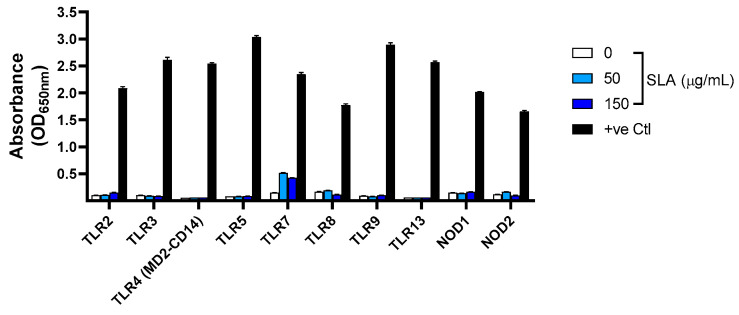
Stimulation of Pattern Recognition Receptors (PRRs) by SLA. HEK-293 cells expressing various PRRs were incubated with SLA or positive control stimulants. Expression of the reporter gene SEAP through receptor-mediated NF-κB activation was determined through spectrophotometry on the following day. Data are presented as mean + SEM (*n* = 3).

As the molecular mechanism of action of SLA is currently unknown, we assayed the ability of SLA to directly stimulate a panel of mouse PRRs in vitro. SLA was incubated at different concentrations (50 and 150 µg/mL) on HEK293 cell lines engineered to express different TLRs or NLRs. Receptor stimulation (Figure 1), as measured through the production of SEAP, revealed that SLA did not induce any activation of TLR2, TLR3, TLR4 (MD2-CD14), TLR5, TLR8, TLR9, TLR13, NOD1 or NOD2. Low levels of activation were seen with TLR7 (OD650 nm of ~0.4–0.5) at both of the tested SLA concentrations. This signal was ~5-fold lower than for the positive control TLR7 agonist CL307.

### 3.2. Activity of SLA Combination Adjuvant Formulations on Humoral Antigen-Specific Antibody Responses 

**Figure 2:**
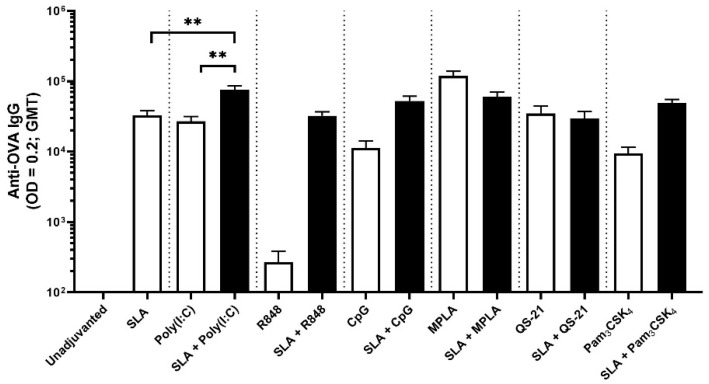
OVA-specific antibody titers in mice. Mice were immunized with OVA antigen alone or formulated with SLA archaeosomes, other adjuvants (Poly(I:C), R848, CpG, MPLA, QS-21 and Pam_3_CSK_4_) or SLA in combination with other adjuvants on days 0 and 21. On day 28 (7 days post-2nd immunization), serum was collected and levels of antigen-specific IgG antibodies measured by ELISA. Grouped data are presented as geometric mean titer + 95% confidence interval (*n* = 10/group). ** represents *p* < 0.01.

As SLA does not appear to strongly activate any of the TLRs tested, we evaluated whether it would synergize with various immunostimulants that act through PRRs, as well as with QS-21, an immunostimulant found in commercial vaccines. Following immunization of mice on days 0 and 21 with OVA alone or OVA formulated with SLA archaeosomes, other adjuvants (Poly(I:C), R848, CpG, MPLA, QS-21 and Pam_3_CSK_4_) or SLA in combination with other adjuvants, antigen-specific IgG responses were assessed in the serum of the immunized mice (day 28, 7 days post-2nd dose). Anti-OVA IgG titers were >2-fold higher in animals receiving formulations adjuvanted with SLA + Poly(I:C) as compared to formulations containing SLA or Poly(I:C) alone (Figure 2; *p* < 0.01). Geomean titers (GMT; lower-upper 95% confidence intervals (CI)) with OVA/SLA + Poly(I:C) were 69,687 (52,036–93,325) vs. 27,148 (16,294–45,233) and 22,400 (13,580–36,948) with SLA and Poly(I:C)-adjuvanted formulations, respectively. Less pronounced increases in anti-OVA IgG titers were also seen in animals immunized with SLA + CpG or SLA + Pam_3_CSK_4_ adjuvanted formulations as compared to the single adjuvant formulations, but they did not reach a level of statistical significance when compared to SLA alone. Analysis of the IgG subtypes showed that SLA as adjuvant induced high levels of IgG1, which were not increased significantly when SLA was combined with any of the other adjuvants. In contrast, the combination of SLA with Poly(I:C), CpG or R848 induced IgG2c antibody levels greater than either adjuvant alone (Figure S1).

### 3.3. Impact of SLA Adjuvant Combination Formulations on Antigen-Specific Cell-Mediated Immunity 

**Figure 3:**
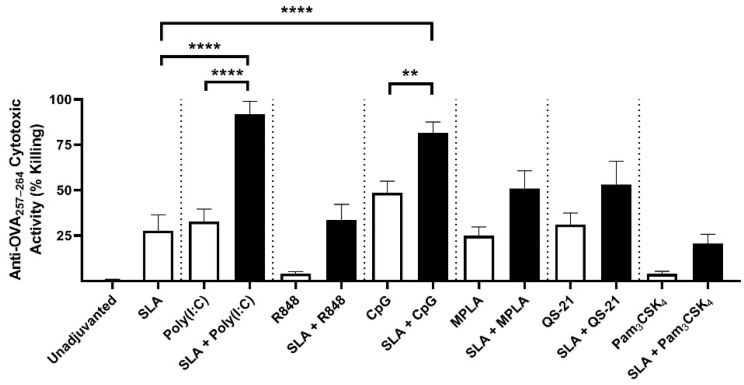
In vivo CTL activity induced by adjuvant combination vaccine formulations. Mice were immunized with vaccine formulations containing OVA antigen alone or formulated with SLA archaeosomes, other adjuvants (Poly(I:C), R848, CpG, MPLA, QS-21, Pam_3_CSK_4_) or SLA in combination with other adjuvants. Target cells were formed by pulsing CFSE-labeled splenocytes from naïve mice with CD8 epitopes from OVA and then transferred to immunized mice. On the following day (day 28: 7 days post-2nd immunization), splenocytes were collected and the levels of the target cells determined by flow cytometry. Grouped data are presented as mean + SEM (*n* = 10/group). ** represents *p* < 0.01 and **** represents *p* < 0.0001.

On day 28, 7 days post-2nd vaccine dose, cellular responses were also assessed in the splenocytes of the immunized mice above. Only background levels of CTL activity (as measured by % killing of SIINFEKL-labeled cells) were measured using OVA alone or in combination with R848 or Pam_3_CSK_4_, whereas low CTL activity was measured with all other single adjuvanted formulations (Figure 3). For example, SLA, Poly(I:C), MPLA and QS-21 alone all had <35% mean cytotoxic activity (% killing), and CpG alone was ~50%. The use of SLA in combination with R848 or Pam_3_CSK_4_ as adjuvant did not result in an increase in CTL activity compared to SLA alone. Although there appeared to be some additive effects when SLA was combined with MPLA or QS-21, the differences were not statistically significant. In contrast, when SLA was combined with either Poly(I:C) or CpG, a significant increase in CTL activity (an average of >80% killing of SIINFEKL-labeled cells) was measured (*p* < 0.01, compared to either adjuvant alone).

**Figure 4:**
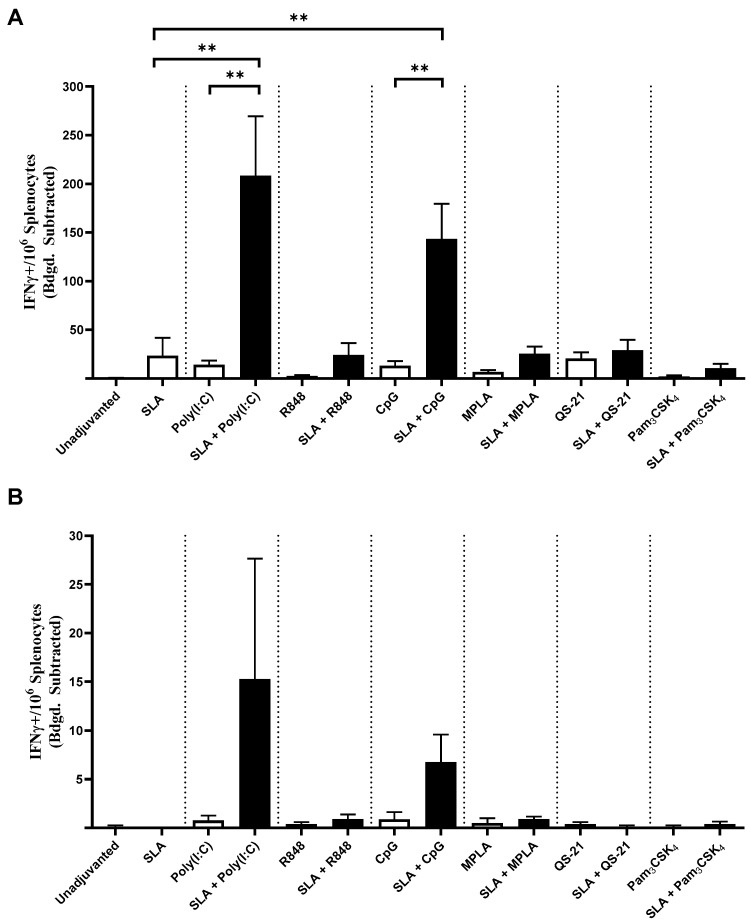
OVA-specific T cells as determined by ELISpot. Splenocytes of mice immunized with OVA antigen alone or formulated with SLA archaeosomes, other adjuvants (Poly(I:C), R848, CpG, MPLA, QS-21, Pam_3_CSK_4_) or SLA in combination with other adjuvants were collected on day 28 (7 days post-2nd immunization) and analyzed by IFN-γ ELISpot when stimulated by CD8 peptide (**A**) or CD4 peptide (**B**). Grouped data are presented as mean + SEM (*n* = 10/group). ** represents *p* < 0.01.

ELISpot was also used to enumerate the number of Ag-specific CD8 (OVA257-264-specific) or CD4 (OVA323-339-specific) T cells in the splenocytes of mice following immunization with the various OVA-containing vaccine formulations above. In accordance with our in vivo CTL results, mice immunized with OVA formulations containing SLA + Poly(I:C) and SLA + CpG showed the highest number of CD8 T cells; an average of 208 and 143 IFNγ+ SIINFEKL-specific spot-forming cell (SFC)/10^6^ splenocytes was observed, respectively (Figure 4A), which was significantly higher (*p* < 0.01) than those in animals immunized using single-adjuvant formulations. All formulations containing single adjuvants or SLA in combination with R848, MPLA, QS-21 or Pam_3_CSK_4_ had low levels of SIINFEKL-specific cells (i.e., <30 IFNγ+ SFC/10^6^ splenocytes). A similar trend was observed when measuring SFCs reactive to the OVA CD4 epitope, where only mice immunized with OVA formulations containing SLA + Poly(I:C) or SLA + CpG had detectable responses (Figure 4B), although these differences were not statistically significant when compared to responses obtained with OVA alone.

### 3.4. Impact of SLA Adjuvant Combination Formulations on HBsAg-Specific Immune Responses

**Figure 5:**
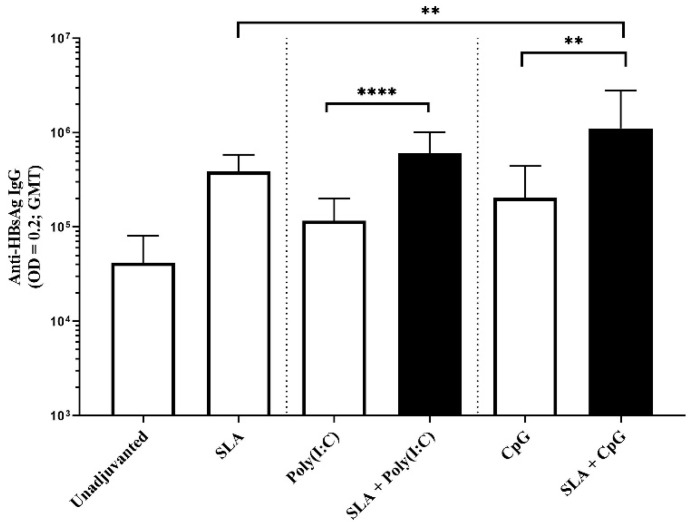
HBsAg-specific antibody titers in mice. Mice were immunized with HBsAg antigen alone or formulated with SLA archaeosomes, other adjuvants ((Poly(I:C) and CpG) or SLA in combination with other adjuvants on days 0 and 21. On day 28 (7 days post-2nd immunization) serum was collected and levels of antigen-specific IgG antibodies measured by ELISA. Grouped data are presented as geometric mean titer + 95% confidence interval (*n* = 5/group). ** represents *p* < 0.01 and **** represents *p* < 0.0001.

To evaluate whether the synergy observed between SLA and Poly(I:C) or CpG would translate to a different antigen model, mice were immunized on days 0 and 21 with HBsAg alone or in combination with SLA, Poly(I:C), CpG, SLA + Poly(I:C) or SLA + CpG. These combinations were selected because they appeared to be the most promising with OVA antigen. The synergistic enhancement in antigen-specific IgG responses was confirmed in HBsAg-immunized mice. Vaccine formulations containing SLA + Poly(I:C) or SLA + CpG induced 1.5 to 3.5-fold higher anti-HBsAg IgG titers than when only a single adjuvant was used (Figure 5).

**Figure 6:**
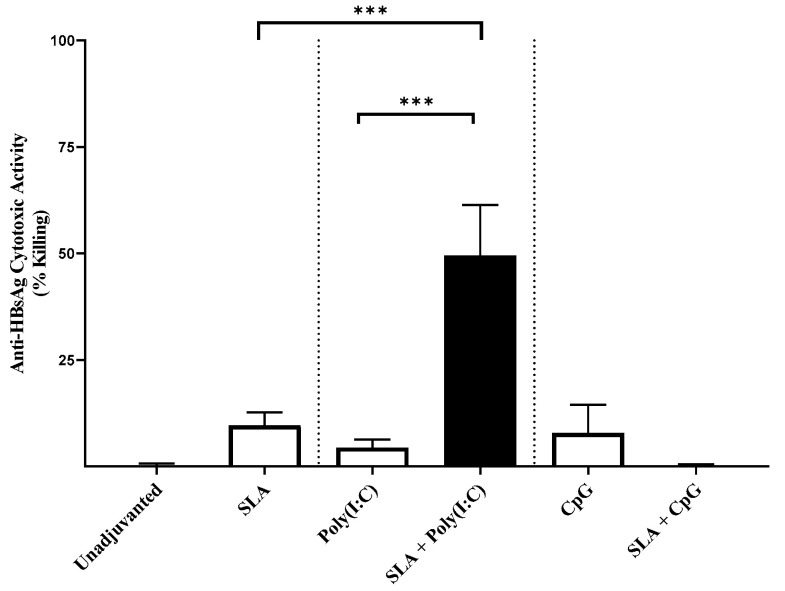
In vivo CTL activity induced by adjuvant combination vaccine formulations. Mice were immunized with vaccine formulations containing HBsAg antigen, alone or formulated with SLA archaeosomes, other adjuvants (Poly(I:C) and CpG) or SLA in combination with other adjuvants. Target cells were formed by pulsing CFSE-labeled splenocytes from naïve mice with CD8 epitopes from HBsAg and then transferred to immunized mice. On the following day (day 28: 7 days post-2nd immunization), splenocytes were collected and the levels of the target cells determined by flow cytometry. Grouped data are presented as mean + SEM (*n* = 5/group). *** represents *p* < 0.001.

While GMT with HBsAg adjuvanted with SLA, Poly(I:C) or CpG were in the range of 116,453 to 386,739, SLA + Poly(I:C) and SLA + CpG adjuvanted formulations induced GMT (lower-upper 95% CI) of 602,427 (359,251 to 1,010,208) and 1,114,971 (444,526 to 2,796,600), respectively. Strong CTL activity was also observed with the SLA + Poly(I:C) adjuvanted formulation (~50% killing following injection of mice with cells labeled with the HBsAg CD8 epitope IPQSLDSWWTSL), compared to <10% killing following immunization with HBsAg adjuvanted with either SLA or Poly(I:C) alone (Figure 6; *p* < 0.001). Interestingly, CTL activity was not increased when SLA was combined with CpG in the HBsAg model, highlighting the importance of selecting different adjuvants or adjuvant combinations for each particular antigen.

## 4. Discussion

In contrast to traditional whole killed or attenuated viral vaccines, subunit vaccines have a more defined composition that is often linked to lower immunogenicity and lack of cell-mediated immunity against intracellular pathogens [26]. To compensate for this, adjuvants are often added to enhance antigen specific humoral and cellular immune responses [26,27]. Traditionally, aluminum salts were added to vaccines to increase immunogenicity. However, in recent years, additional stand-alone adjuvants (e.g., CpG, MF-59 and AS03) and adjuvant combinations (e.g., AS01 and AS04) have also been included in approved vaccines. However, as these are not always readily available or appropriate for certain indications, the need for strong novel adjuvants remains.

We have previously demonstrated that SLA archaeosomes are capable of enhancing both humoral and cell-mediated immune activity when used as an adjuvant with multiple antigens: OVA, HBsAg [15], hepatitis C virus E1/E2 envelope protein [17], H1N1 influenza hemagglutinin protein (18), melanoma cancer [19] and *Schistosoma mansoni* Cathepsin B [16]. Furthermore, using OVA or HBsAg as antigens, we also demonstrated that SLA archaeosomes induce equivalent or superior antigen-specific immune responses to those obtained with many other adjuvants, including TLR3/4/9 agonists, oil-in-water and water-in-oil emulsions and aluminum hydroxide [15]. Initial studies indicated that SLA archaeosomes enhance immune cell infiltration, antigen retention and antigen uptake at the injection site [22]. However, no studies to date have evaluated the use of SLA archaeosomes, or to the best of our knowledge any other type of archaeosome, in combination with a panel of other types of adjuvants. Therefore, we sought to evaluate whether the adjuvanticity of SLA-based archaeosome adjuvant formulations could be further enhanced by combining with other adjuvants. We first demonstrated that SLA archaeosomes did not strongly activate Toll-like receptor signalling pathways directly and as such, due to their differing mechanisms of action, could potentially synergize with TLR agonists when co-formulated together. Five TLR agonists, targeting various TLRs, namely, the TLR1/2 agonist Pam_3_CSK_4_, the TLR3 agonist poly(I:C), the TLR4 agonist MPLA, the TLR 7/8 agonist R848, the TLR9 agonist CpG as well as the saponin QS-21, were selected and combined with SLA archaeosomes to compare their ability to generate antigen-specific humoral and cellular immune responses. The strongest synergy was observed between SLA archaeosomes and either CpG or Poly(I:C) in both humoral (Ag-specific IgG) and cell-mediated (antigen-dependent cytotoxicity and IFN-γ production) immune readouts with slight differences observed between the antigens tested. This may have been due to the different nature of the antigens, namely, soluble protein (OVA) versus virus-like particles (HBsAg). Although in most cases, differences were not significant, an additive effect on the increase in antigen-specific IgG antibody was also measured when mice were immunized with the combination of SLA archaeosome with Pam_3_CSK_4_ using OVA antigen, as well as Poly(I:C) using HBsAg. It is possible that additional synergies or additive effects could have been observed had different adjuvant doses also been evaluated. 

The synergistic effect of SLA archaeosomes with CpG or Poly(I:C) was particularly strong with both antigens. This was surprising, as although multiple groups have shown that liposomes can be used to enhance CpG or Poly(I:C)-mediated effects, in those studies, the TLR agonists were generally co-encapsulated along with the antigen within liposomes rather than admixed [8,9,28]. In those cases, the liposome was used to protect the antigen and/or prolong the half-life of the adjuvant, lacking any inherent immunostimulatory effects when used on its own. Although in the current study we did not co-localize antigens, CpG or Poly(I:C) within the vaccine formulations to determine whether one or more components were associated with the archaeosomes, we previously showed using cryogenic transmission electron microscopy that in contrast to archaeosomes prepared using a conventional entrapment process, most of the antigens in an admixed formulation were observed in free form outside of vesicles [21]. Unlike most cationic liposomes used to deliver negatively charged nucleic acids, SLA archaeosomes are composed of a negatively charged lipid and hence are unlikely to bind to either CpG or Poly(I:C) directly. However, while it is unlikely that CpG or Poly(I:C) would interact with archaeosomes based on charge, it cannot be ruled out that the presence of archaeosomes could impact adjuvant distribution as has been shown previously for antigens [22]. 

In our studies, we found stronger synergy between SLA and CpG or Poly(I:C) than with other TLR agonists such as R848, MPLA or Pam_3_CSK_4_. It is possible that this is a result of either TLR location or relative adjuvant sizes. Mammalian TLRs can be divided into two subgroups—those that are located extracellularly and recognize microbial cell surface components, such as lipopolysaccharide (TLR4), flagellin (TLR5) and bacterial lipoproteins (TLRs 1, 2 and 6), and those that are found intracellularly in endosomes and detect nucleic acids, such as double-stranded RNA (TLR3), single-stranded RNA (TLR 7 and 8), unmethylated DNA containing CpG motifs (TLR9) and bacterial ribosomal RNA (TLR13) [29]. In our study, we saw the strongest synergy with two intracellular TLR agonists, namely, CpG (TLR9 agonist) and Poly(I:C) (TLR3 agonist), and much weaker or no additive effects with the extracellular TLR agonists, namely, Pam_3_CSK_4_ (TLR1/2 agonist) and MPLA (TLR4 agonist). However, there were no additive effects with the addition of R848 (an agonist for the intracellular TLR7/8) to SLA archaeosomes. It is possible that this is due to the relative differences in molecular weight between R848 (314.4 g/mol) and CpG (ODN 1826, B class; 6364 g/mol) or Poly(I:C) (HMW; >10^6^ g/mol) leading to a more rapid migration of R848 away from the injection site, or it could be that R848 is simply a much weaker adjuvant than CpG or Poly(I:C) [30]. This would need to be further studied using additional TLR 7/8 agonists, such as ssRNA, but challenges, such as the inherent instability of ssRNA and its relative inability to readily enter the cell, would need to be addressed. It is also worth noting that we used suboptimal doses of adjuvants in this study in order to better detect any synergistic responses. It is possible that synergy patterns may have been different if higher doses were used.

CpG has also been shown to synergize with aluminum salts and to redirect pre-established Th2 responses into a more mixed or Th1-biased immune response [31]. Poly(I:C) combinations have also previously been reported, including with aluminum salts [32] and liposomes. While those studies combined a Th1-biased (i.e., TLR agonist) and a Th2-biased adjuvant (i.e., aluminum salts), in our studies, we observed a synergy between two adjuvant platforms each capable of inducing Th1-associated cytotoxic responses on their own. This may be due to the fact that these adjuvants rely on different pathways to induce Th1-biased immune responses. The nature of the antigen and the mouse model may also influence the ability of the adjuvants to synergize. While we saw formulations containing SLA archaeosomes and CpG inducing stronger anti-OVA cytotoxic responses, this synergy was largely absent in our HBsAg model. We previously showed a similar trend with CpG + aluminum adjuvant formulation, where the combination strongly enhanced CTL responses to OVA but not HBsAg [15]. 

QS-21 is a triterpene glycoside purified from the bark extract of *Quillaja saponaria Molina* and is one of the components in AS01 (a liposome-based vaccine adjuvant system containing the TLR4 agonist, MPLA and the saponin QS-21), which is used in the shingles vaccine, Shingrix and many other vaccines currently in development. When used alone, QS-21 has its own inherent adjuvant properties, which are enhanced when co-administered with the TLR4 agonist, MPLA. In AS01, liposomes are used to decrease the hemolytic activity associated with QS-21 [2]. Since SLA archaeosomes are a form of liposomes which also possess strong adjuvant activity, we wanted to evaluate whether SLA archaeosomes would synergize with either the QS-21 or the MPLA components of AS01. However, co-administration of SLA archaeosomes with either MPLA or QS-21 did not induce a significant enhancement of either humoral or cell-mediated immune responses. It is possible that if we had evaluated SLA in combination with both MPLA and QS-21, as contained in AS01, we would have better results, but this was out of the scope of the current study. It is also possible that if one or both of these adjuvants had been incorporated with SLA into liposomes rather than simply admixed, a synergy would have been observed. For example, MPLA would likely have been anchored into the lipid membrane of the liposomes if present during their formation. However, since our goal was to evaluate simple admixed formulations, this was not evaluated. Although no obvious negative effects were observed in this study, an additional more in-depth evaluation of any potential local and systemic toxicities associated with the adjuvant combinations would be an important next step. Additional studies could also include a detailed comparison of Th1, Th2 and Th17 responses with the different adjuvant combinations. 

In summary, the toolbox for vaccine development can be extended by combining adjuvants with different mechanisms of action. In this regard, our results demonstrate that the combined adjuvant of SLA + Poly(I:C) and SLA + CpG are effective in stimulating both humoral and cellular immune responses. Moreover, the combination of SLA + Poly(I:C) and SLA + CpG not only strengthens the stimulated antigen-specific immune responses but also modulates them in the direction of a Th1 response.

## 5. Conclusions

When used as an adjuvant, SLA archaeosomes alone can induce strong humoral and cell-mediated immune responses to co-administered antigen. Herein, we showed that these responses can be greatly increased if SLA archaeosomes are combined with either Poly(I:C) or CpG. Overall, this study expands the utility of SLA archaeosome adjuvants by showing that they can be combined with TLRs, which could be used to significantly improve the efficacy of subunit vaccines.

## Figures and Tables

**Table 1:** Vaccine formulations using model antigen ovalbumin (OVA) as antigen.

Group No. (*n* = 10)	Adjuvant	Adjuvant Dose µg/50 µL
1	None (OVA alone)	-
2	SLA	500
3	R848	5
4	CpG	5
5	MPLA	5
6	QS-21	5
7	Pam_3_CSK_4_	5
8	Poly(I:C)	20
9	SLA + R848	500 + 5
10	SLA + CpG	500 + 5
11	SLA + MPLA	500 + 5
12	SLA + QS-21	500 + 5
13	SLA + Pam_3_CSK_4_	500 + 5
14	SLA + Poly(I:C)	500 + 20

**Table 2:** Vaccine formulations using Hepatitis B surface antigen (HBsAg) as antigen.

Group No. (*n* = 5)	Adjuvant	Adjuvant Dose µg/50 µL
1	None (HBsAg alone)	-
2	SLA	1000
3	Poly(I:C)	40
4	SLA + Poly(I:C)	1000 + 40
5	CpG	10
6	SLA + CpG	1000 + 10

## Data Availability

The data presented in this study are available on request from the corresponding author.

## Supplementary Materials

The following are available online at https://www.mdpi.com/1999-4923/13/2/205/s1, Figure S1: OVA-specific antibody titers in mice. Mice were immunized with OVA antigen alone or formulated with SLA archaeosomes, other adjuvants (Poly(I:C), R848, CpG, MPLA, QS-21 and Pam_3_CSK_4_) or SLA in combination with other adjuvants on days 0 & 21. On Day 28 (7 days post 2nd immunization) serum was collected and levels of antigen-specific IgG1 and IgG2c antibodies measured by ELISA. Grouped data is presented as geometric mean titer + 95% Confidence Interval (*n* = 10/group). * *p* < 0.05, ** *p* < 0.01, *** *p* < 0.001 and **** *p* < 0.0001.
