# Supplementary material for: The Synergistic Effects of Sulfated Lactosyl Archaeol Archaeosomes When Combined with Different Adjuvants in a Murine Model
Source: Pharmaceutics. 2021 Feb 2;13(2):205. doi: 10.3390/pharmaceutics13020205 (PMC7913188; doi:10.3390/pharmaceutics13020205)
Supplement: Supplementary file 1 [file pharmaceutics-13-00205-s001.pdf]

pharmaceutics

MDPI

Supplementary Materials: The Synergistic Effects of Sulfated Lactosyl Archaeol Archaeosomes when Combined with Different Adjuvants in a Murine Model

Yimei Jia, Bassel Akache, Gerard Agbayani, Vandana Chandan, Renu Dudani, Blair A. Harrison, Lise Deschatelets, Usha D. Hemraz, Edmond Lam, Sophie Régnier, Felicity C. Stark, Lakshmi Krishnan, Michael J. McCluskie

Citation: Jia, Y.; Akache, B.; Agbayani, G.; Chandan, V.; Dudani, R.; Harrison, B.A.; Deschatelets, L.; Hemraz, U.D.; Lam, E.; Régnier, S.; et al. The Synergistic Effects of Sulfated Lactosyl Archaeol Archaeosomes When Combined with Different Adjuvants in a Murine Model. *Pharmaceutics* **2021**, *13*, 205. https://doi.org/10.3390/pharmaceutics13020205

Academic Editor: Nirmal Marasini
Received: 18 January 2021
Accepted: 28 January 2021
Published: 2 February 2021

Publisher's Note: MDPI stays neutral with regard to jurisdictional claims in published maps and institutional affiliations.

Copyright: © 2021 by the authors. Licensee MDPI, Basel, Switzerland. This article is an open access article distributed under the terms and conditions of the Creative Commons Attribution (CC BY) license (http://creativecommons.org/licenses/by/4.0/).

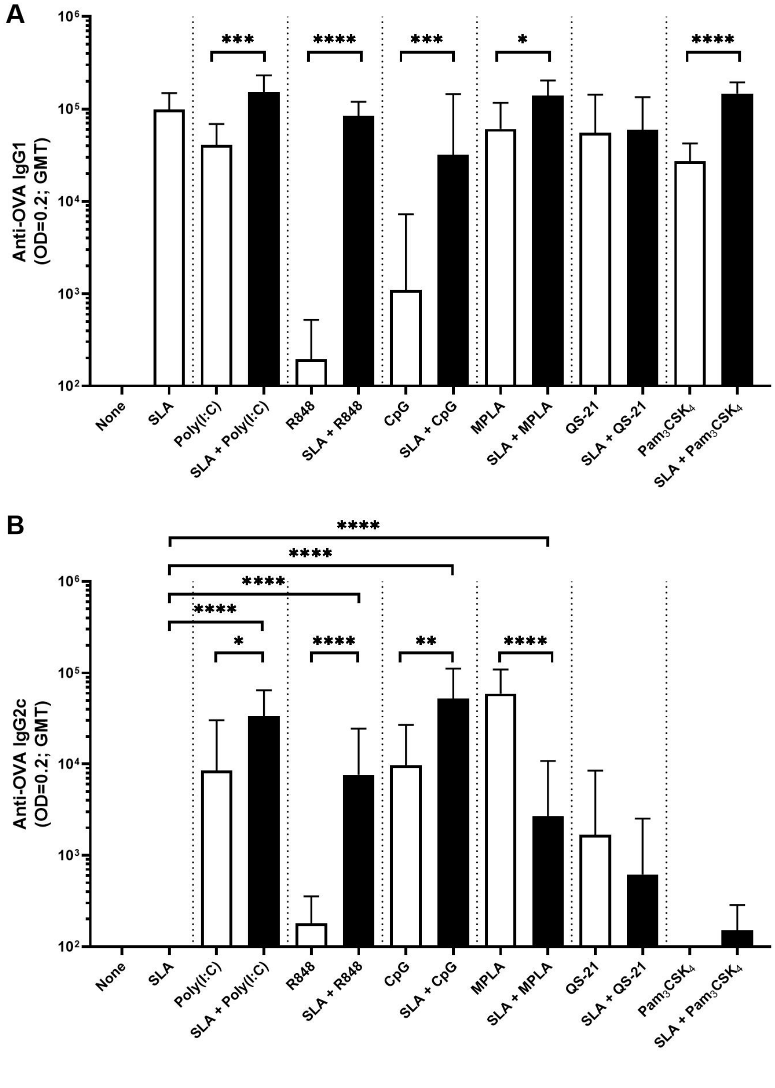

Figure S1. OVA-specific antibody titers in mice. Mice were immunized with OVA antigen alone or formulated with SLA archaeosomes, other adjuvants (Poly(I:C), R848, CpG, MPLA, QS-21 and Pam_3CSK_4) or SLA in combination with other adjuvants on days 0 & 21. On Day 28 (7 days post 2nd immunization) serum was collected and levels of antigen-specific IgG1 and IgG2c antibodies measured by ELISA. Grouped data is presented as geometric mean titer + 95% Confidence Interval (n = 10/group). * $p < 0.05$, ** $p < 0.01$, *** $p < 0.001$ and **** $p < 0.0001$.
